# Proinflammatory role of inducible nitric oxide synthase in acute hyperoxic lung injury

**DOI:** 10.1186/1465-9921-5-11

**Published:** 2004-09-15

**Authors:** Anne-Karin Hesse, Martina Dörger, Christian Kupatt, Fritz Krombach

**Affiliations:** 1 Institute for Surgical Research, University of Munich, Marchioninistr. 15, 81366 Munich, Germany; 2 Department of Internal Medicine I, University of Munich, Marchioninistr. 15, 81366 Munich, Germany

## Abstract

**Background:**

Hyperoxic exposures are often found in clinical settings of respiratory insufficient patients, although oxygen therapy (>50% O_2_) can result in the development of acute hyperoxic lung injury within a few days. Upon hyperoxic exposure, the inducible nitric oxide synthase (iNOS) is activated by a variety of proinflammatory cytokines both *in vitro *and *in vivo*. In the present study, we used a murine hyperoxic model to evaluate the effects of iNOS deficiency on the inflammatory response.

**Methods:**

Wild-type and iNOS-deficient mice were exposed to normoxia, 60% O_2 _or >95% O_2 _for 72 h.

**Results:**

Exposure to >95% O_2 _resulted in an increased iNOS mRNA and protein expression in the lungs from wild-type mice. No significant effects of iNOS deficiency on cell differential in bronchoalveolar lavage fluid were observed. However, hyperoxia induced a significant increase in total cell count, protein concentration, LDH activity, lipid peroxidation, and TNF-α concentration in the bronchoalveolar lavage fluid compared to iNOS knockout mice. Moreover, binding activity of NF-κB and AP-1 appeared to be higher in wild-type than in iNOS-deficient mice.

**Conclusion:**

Taken together, our results provide evidence to suggest that iNOS plays a proinflammatory role in acute hyperoxic lung injury.

## Background

Supplemental oxygen therapy is administered for the treatment of tissue hypoxia, most commonly in an intensive care setting of respiratory insufficient patients, though its potent toxicity is well described [1]. The pathophysiology of oxygen injury is characterized by lung inflammation including activation and recruitment of neutrophils and alveolar macrophages, tissue and alveolar edema, surfactant dysfunction, and excess production of free radicals and inflammatory cytokines [2-4]. Although the exact mechanisms of pulmonary oxygen toxicity are still unknown, compelling evidence suggests that reactive oxygen species such as superoxide anion, hydroxyl radical, and hydrogen peroxide are important mediators of lung injury [5-7]. High oxygen concentrations induce cellular damage by several mechanisms such as oxidation of proteins, peroxidation of membrane lipids, and breakage of DNA strands [8-10]. Moreover, hyperoxia also induces the release of a wide spectrum of proinflammatory cytokines such as tumor necrosis factor-[11-13]. However, the precise molecular mechanisms by which hyperoxia produces acute lung injury remain unclear.

Reactive oxygen species can also react with other free radicals such as nitric oxide (NO) to yield more cytotoxic species including peroxynitrite anion [14,15]. Peroxynitrite is a strong oxidizing agent that can also initiate lipid peroxidation [16,17]. Since the discovery of NO as a potent vascular smooth muscle relaxant and regulator of blood pressure, NO generated by the inducible nitric oxide synthase (iNOS) has been identified in many cell types such as alveolar macrophages or epithelial cells and implicated in a variety of biological roles [15,18,19].

NO is synthesized from L-arginine by two main isoforms of the NO synthase: the constitutive and the inducible isoform [20]. The NOS enzymes are complex homodimeric proteins consisting of a N-terminal oxygenase domain, a central calmodulin binding sequence, and a C-terminal reductase domain [21,22]. Inducible NOS is expressed following induction by a variety of inflammatory cytokines such as TNF-α [22] or by lipopolysaccharide (LPS) [23-25]. Constitutive iNOS expression has been reported in the lung [26,27] and several inflammatory processes involving the lung, such as sepsis [23-25], asbestosis-induced lung injury [28,29] and hyperoxia [30,31] are associated with an elevated NO production. However, the effect of hyperoxia on endogenous NO production is a matter of controversial discussion, depending on the experimental conditions [30,32-34].

Inappropriate regulation of nuclear factor-κB (NF-κB)-and activator protein-1 (AP-1)-mediated transcription has also been associated with pathological conditions, including acute inflammation such as hyperoxic exposure [35]. Intracellular signaling pathways leading to an activation of transcriptional regulators such as NF-κB and AP-1 can be affected by reactive oxygen and nitrogen species [36-38]. Both transcription factors are activated in lung cells after short periods of hyperoxic exposure. Binding sites for NF-κB and AP-1 are present in the promotor of the iNOS gene and of proinflammatory cytokines such as TNF-α [35,39,40].

The objective of this study was to investigate the effect of iNOS deficiency on acute hyperoxic lung injury. As indicators of lung hyperpermeability, lavageable lung protein and LDH activity were measured; lung lipid oxidation was assessed based on the levels of thiobarbituric acid reactive substances. To characterize the inflammatory process, lavageable cell counts, cell differential, and TNF- concentration were analyzed. Binding activity of NF-κB and AP-1 was investigated in order to elucidate transcriptional mechanisms for iNOS and TNF-α expression.

## Methods

### Animals

Inducible NOS-deficient mice (C57BL6/J × 129SvEv) were originally provided by Dr. J. Mudgett (Merck & Co., Rathway, New Jersey, USA), Dr. J. MacMicking, and Dr. C. Nathan (Cornell University Medical College, New York, USA) [41]. As controls, matching wild-type mice were used (C57BL6/J × 129SvEv). Animals were bred in the facilities of the Institute for Surgical Research (Munich, Germany). Protocols used in this study were approved by the appropriate government body.

### Hyperoxic exposure

Male mice (12 – 16 weeks old, body weight between 26.1 g and 27.3 g) were kept in groups of seven in a sealed Plexiglas chamber (27 × 27 × 20 cm^3^). Animals were randomized and exposed to 60% O_2 _and >95% O_2 _with a gas flow rate of 6 l/min at atmospheric pressure for 72 h. Mice exposed to room air in the same chamber served as controls. O_2 _levels were monitored twice daily with an oxygen analyzer (Drägerwerk AG, Lübeck, Germany). The environmental temperature was maintained at 24°C ± 1, relative humidity was 73% ± 13, and air pressure was 947 mbar ± 5. Oxygen was humidified by bubbling through a water chamber. The Plexiglas chamber bottom was lined with soda lime for CO_2 _absorption (Mallinkrodt Baker B. V., Deventer, Holland). Exposures were continuous for the time indicated except for 5 – 10 min daily when the chamber was opened for housekeeping purposes. Animals were kept on a 12 h light/dark cycle. Standard rodent food and water were available *ad libitum*.

### Bronchoalveolar lavage cell counts and cell differential

Immediately following exposure, mice were anaesthetized by intraperitoneal injection of sodium pentobarbital (10 mg/kg body weight, Narcoren^®^, Merial, Halbergmoos, Germany). Tracheotomy was performed and a 20 G × 32 mm needle (Abbocath^®^-T, Venisystems, Sligo, Ireland) was inserted and secured. Bronchoalveolar lavage (BAL) was performed five times with 1 ml of sterile non-pyrogenic phosphate-buffered saline solution (PBS; Serva, Heidelberg, Germany) in each animal. After centrifugation at 300 × g for 10 min, the supernatant was collected and stored at -20°C and -80°C for later protein assays. The BAL cell pellet was resuspended in PBS and washed by centrifugation. Cells were stained with May-Grunwald-Giemsa (Varistain 4, Shandon Labortechnik GmbH, Frankfurt, Germany) to identify cellular populations. Total cell counts were assessed with a hemacytometer (Coulter Ac T 8, Coulter Electronics, Krefeld, Germany).

### Lavageable lung protein assay

Cell free BAL fluid was evaluated for total protein content by the bicinchoninic acid assay using bovine serum albumin (PAA Laboratories, Linz, Austria) based on a method of Smith et al. [42].

### Lactate dehydrogenase activity assay

To evaluate lactate dehydrogenase (LDH) activity in cell free BAL fluids, a commercially available kit was used (LDH Optimiert, Roche Diagnostics, Mannheim, Germany).

### TNF-α assay

Concentration of TNF-α in cell free BAL fluid was measured by an enzyme linked immunosorbent assay using a commercially available kit (EM-TNFA, Endogen, Woburn, Massachusetts, USA). Briefly, 50 ml biotinylated antibody reagent were added to 50 ml-samples in an anti-mouse TNF-α pre-coated strip well plate. After incubation for 2 h at room temperature, the plate was washed, a streptavidin horseradish peroxidase solution and the 3, 3',5, 5'-tetramethylbenzidine substrate solution were added and incubated in the dark. The absorbance was detected at 450 nm in a microplate reader (EAR 400 AT, Salzburger Labortechnik, Salzburg, Austria). A standard curve was used to determine the amount of TNF-α concentration in the samples.

### Reverse transcriptase-polymerase chain reaction

Total RNA was isolated from non-lavaged lung homogenate of each mouse (RNeasy Mini Kit, Quiagen, Hilden, Germany), reverse transcribed into cDNA in a volume of 20 ml, containing 2 μg RNA, 1.5 μM Oligo-p(dT)15-primer, 5 × PCR-buffer, 0.1 M DTT, 10 nM dNTP-mix and 200 U/μl of Moloney murine leukemia virus reverse transcriptase. Reverse transcriptase-polymerase chain reaction (RT-PCR) amplifications were performed with aliquots of cDNA (3 μl) in total volume of 50 μl (5 μl 10 × PCR reaction-buffer, 1 μl dNTP-mix, 1 μl each of forward and reverse single strand DNA primers specific for mouse iNOS, 38.8 μl sterile deionized water, 0.2 μl Taq DNA-polymerase 1 U/ml). Oligonucleotide primers for iNOS were 5'-CAC AAG GCC ACA TCG GAT TTC-3' (sense) and 5'-TGC ATA CCA CTT CAA CCC AG-3' (antisense). Co-amplification of the housekeeping gene β-actin served as an internal control, using the following primers, 5'-GGA CTC CTA TGT GGG TGA CGA GG-3' (sense), 5'-GGG AGA GCA TAG CCC TCG TAG AT-3' (antisense). RT-PCR was started with 1 min incubation at 95°C followed by the steps of denaturation at 95°C for 45 sec, annealing at 55 – 64°C for 45 sec, elongation at 72°C for 1 min. The number of cycles (30 – 35 each) was chosen to ensure that the amplification product did not reach the level of saturation. Reactions were electrophoresed in 1% agarose gel and stained with ethidium bromide. The densitometry of each cDNA band was quantified using BIO-1D.V96 software and the ratio of iNOS cDNA to β-actin cDNA was determined.

### Electrophoretic mobility shift assay

Nuclear protein extracts were prepared from pooled lung tissue as previously described [43]. Briefly, the oligonucleotides were incubated with a binding buffer (0.04 M Tris, 0.2 M NaCl, 2 mM EDTA, 8% glycerine, 2 μm Ficoll 400, 0.2 mM PMSF, 4 mM DTT). After 5 min incubation, 5 μ l of [γ^32^P]-dATP end-labeled double-stranded oligonucleotides containing an NF-κB-consensus sequence (5'-AGT TGA GGG GAC TTT CCC AGG C-3') or AP-1-consensus sequence (5'-CGC TTG ATG AGT CAG CCG GAA-3') were added to the reaction followed by an incubation for 1 h at 37°C. The mixture was subjected to electrophoresis on a 6% PAA-Gel (75% H_2_O, 45 mM Tris, 45 mM bore acid, 1 mM EDTA, pH 8. 6% APS, 60 μl TEMED) for 2 h at 250 V.

### Thiobarbituric acid reactive substances assay

Concentration of thiobarbituric acid reactive substances (TBARS) was evaluated with an assay according to Thiery et al. [44]. BAL fluid was prepared to denaturate proteins with 50% trichloroacetic acid. The supernatants were transferred to a clean tube and 75 μl of 1.3% thiobarbituric acid (Sigma Chemie, Deisenhofen, Germany) in 0.3% NaOH were added. After incubation for 1 h at 90°C and subsequent cooling in ice water, samples were centrifuged for 6 min. Finally, 200 μl of sample were transferred to a 96-well plate and the absorbance at 530 nm was read in a microplate reader (Dynex Technologies, Denkendorf, Germany). TBARS were quantified by using a standard curve of malondialdehyde (Sigma Chemie, Germany).

### Statistical analysis

Results are presented as the group mean ± standard error of the mean (SEM). Statistical comparison between values of the three oxygen concentrations was performed by using analysis of variance on ranks and Mann-Whitney rank sum test followed by Bonferroni's correction. Statistical comparison between wild-type and iNOS knockout mice was analyzed by using Mann-Whitney rank sum test. Significance was accepted at p < 0.05.

## Results

### General conditions of the animals and body weight

Wild-type and iNOS knockout mice all survived hyperoxia the entire 72 h. After hyperoxic exposure >95% O_2_, animals showed signs of reduced general conditions and reactions. Hyperoxic exposure >95% O_2 _also caused a significant reduction in body weight of wild-type mice compared to normoxic conditions and 60% oxygen exposure within the 72 h experimental period. In contrast, there was no significant change in body weight of iNOS knockout mice before and after normoxia and hyperoxia, respectively (table 1).

**Table 1:** Body weight (g) of wild-type and iNOS knockout mice after 72 h exposure to 21%, 60%, and >95% O_2_*

		21% O_2_	60% O_2_	>95% O_2_
wild-type mice	before exposure	27.1 ± 1.0	26.5 ± 0.8	27.8 ± 0.9
	after exposure	28.1 ± 0.9	28.1 ± 0.3	23.2 ± 0.6^#^
iNOS knockout mice	before exposure	24.9 ± 0.7	23.3 ± 0.5	25.2 ± 0.9
	after exposure	26.1 ± 0.4	24.0 ± 0.5	24.1 ± 0.7

*Each value represents mean ± SEM, n = 7.

^#^p < 0.05 vs. before exposure.

### Differential and total cell counts

**Figure 1:**
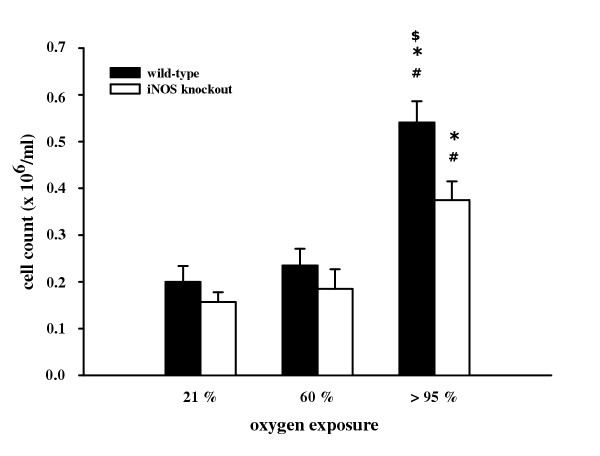
Total cell counts in BAL from wild-type and iNOS knockout mice after 72 h exposure to 21%, 60%, and >95% O_2_. Data are mean ± SEM of seven mice for each group. ^#^p < 0.05 vs. normoxia; *p < 0.05 vs. 60% O_2_; ^$^p < 0.05 vs. iNOS knockout mice.

BAL in wild-type and iNOS knockout mice was performed to assess cellular infiltration in the alveolar space upon 72 h hyperoxic exposure (60% and >95% O_2_). Results presented in table 2 demonstrate no differences in baseline cell differentials between wild-type and iNOS knockout mice. Upon 72 h exposure to >95% O_2_, there was a significant decrease in the percentage of alveolar macrophages as well as a significant increase in the percentage of neutrophils and lymphocytes in both wild-type and iNOS knockout mice compared to normoxic conditions. No significant differences between wild-type- and iNOS knockout mice were found. However, hyperoxic exposure (>95% O_2_) resulted in a significant increase in total BAL cell counts after 72 h in wild-type (0.54 ± 0.05 × 10^6^/ml) and in iNOS knockout mice (0.38 ± 0.04 × 10^6^/ml) compared to normoxia (0.20 ± 0.03 × 10^6^/ml and 0.16 ± 0.02 × 10^6^/ml, respectively) and 60% O_2 _exposure (0.24 ± 0.04 × 10^6^/ml and 0.19 ± 0.04 × 10^6^/ml, respectively). This increase in BAL total cell counts under >95% O_2 _was significantly higher in wild-type than in iNOS knockout mice (figure 1).

**Table 2:** BAL cell differential in wild-type and iNOS knockout mice after 72 h exposure to 21%, 60%, and >95% O_2_*

		alveolar macrophages (%)	neutrophils (%)	lymphocytes (%)
wild-type mice	21% O_2_	98.8 ± 0.5	0.0 ± 0.0	1.2 ± 0.5
	60% O_2_	97.7 ± 1.0	0.3 ± 0.2	2.0 ± 1.0
	>95% O_2_	86.2 ± 1.8#*	4.6 ± 1.0#*	9.2 ± 0.9#*
iNOS knockout mice	21% O_2_	99.0 ± 0.4	0.7 ± 0.2	0.3 ± 0.2
	60% O_2_	98.9 ± 0.5	0.4 ± 0.3	0.7 ± 0.4
	>95% O_2_	85.1 ± 2.0#*	4.9 ± 1.3#*	10.0 ± 1.2#*

*Each value represents mean ± SEM, n = 7.

^#^p < 0.05 vs. normoxia; *p < 0.05 vs. 60% O_2_

### Lavageable lung protein

Total protein concentration in the BAL fluid was determined as an indicator of lung hyperpermeability induced by hyperoxic exposure. Under normoxia and 60% O_2_, total protein concentration did not differ between wild-type mice (21% O_2_: 86.4 ± 37.3 μg/ml; 60% O_2_: 95.5 ± 22.8 μg/ml) and knockout mice (21% O_2_: 157.1 ± 23.7 μg/ml; 60% O_2_: 86.0 ± 26.6 μg/ml). Exposure to >95% O_2 _resulted in a significant increase in protein concentration in wild-type mice (973.8 ± 95.7 μg/ml) and only in a modest increase in iNOS knockout mice (326.8 ± 90.4 μg/ml) that did not reach statistical significance.

### Lactate dehydrogenase activity

**Figure 3:**
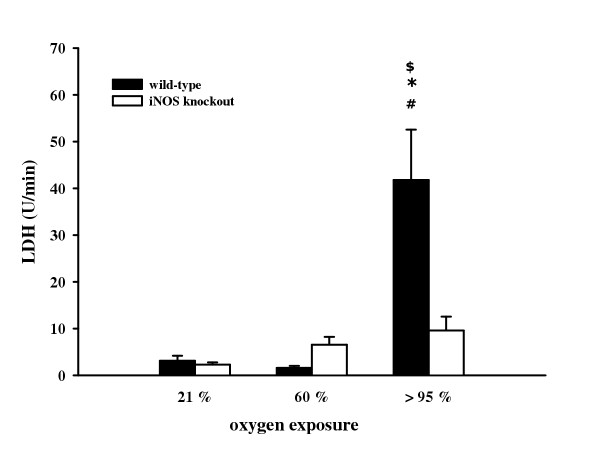
Lactate dehydrogenase activity in BAL from wild-type and iNOS knockout mice after 72 h exposure to 21%, 60%, and >95% O_2_. Data are mean ± SEM of seven mice for each group. ^#^p < 0.05 vs. normoxia; *p < 0.05 vs. 60% O_2_; ^$^p < 0.05 vs. iNOS knockout mice.

As an indicator of cellular damage, LDH activity was measured in BAL fluid. Under normoxic conditions and 60% O_2_, LDH activity was comparable between wild-type (21% O_2_: 3.1 ± 1.0 U/min/ml; 60% O_2_: 1.6 ± 0.4 U/min/ml) and iNOS knockout mice (21% O_2_: 2.3 ± 0.5 U/min/ml; 60% O_2_: 6.5 ± 1.7 U/min/ml). Exposure to >95% O_2 _resulted in a significant enhancement of LDH activity in wild-type mice (41.8 ± 10.8 U/min/ml) compared to iNOS knockout mice (9.6 ± 3.0 U/min/ml) and to normoxia (figure 3).

### TNF-α concentration

**Figure 4:**
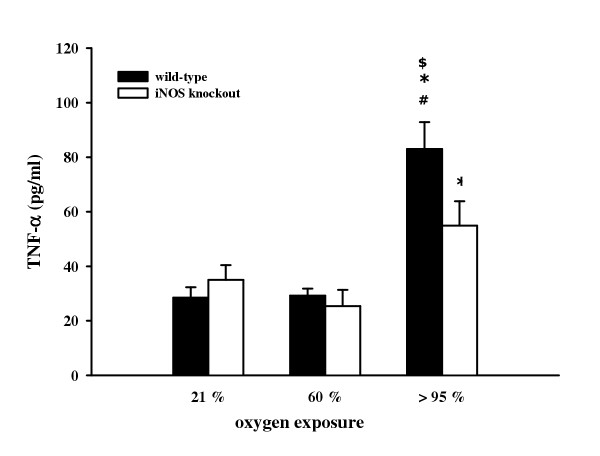
TNF-α concentration in BAL from wild-type and iNOS knockout mice after 72 h exposure to 21%, 60%, and >95% O_2_. Data are mean ± SEM of seven mice for each group. ^#^p < 0.05 vs. normoxia; *p < 0.05 vs. 60% O_2_; ^$^p < 0.05 vs. iNOS knockout mice.

TNF-α concentrations were determined in BAL fluid to investigate inflammatory cytokine release. Under normoxic conditions, TNF-α release did not differ between wild-type (28.5 ± 3.8 pg/ml) and iNOS knockout mice (35.0 ± 5.4 pg/ml), the same as upon 60% O_2 _exposure (29.2 ± 2.6 pg/ml and 25.4 ± 6.0 pg/ml, respectively). However, there was a significantly enhanced TNF-α release measured upon >95% O_2 _exposure in wild-type (83.0 ± 9.8 pg/ml) and iNOS knockout mice (54.9 ± 9.0 pg/ml) compared to normoxic conditions. TNF-α concentration was significantly higher in wild-type than in iNOS knockout animals (figure 4).

### Concentration of thiobarbituric acid reactive substances

**Figure 5:**
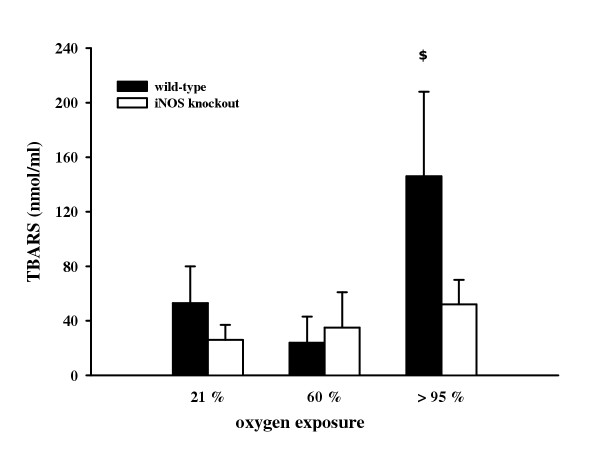
Concentration of thiobarbituric acid reactive substances in BAL from wild-type and iNOS knockout mice after 72 h exposure to 21%, 60%, and >95% O_2_. Data are mean ± SEM of seven mice for each group. ^$^p < 0.05 vs. iNOS knockout mice.

Lung lipid peroxidation was assessed based on the levels of thiobarbituric acid reactive substances in BAL (figure 5). Wild-type mice exposed to >95% O_2 _exhibited a pronounced increase in concentration of TBARS (146.0 ± 62.0 nmol/ml) compared to normoxia (35.0 ± 14.0 nmol/ml) and 60% O_2 _(31.0 ± 17.0 nmol/ml). In iNOS knockout mice, concentrations of TBARS after >95% O_2 _(52.0 ± 18.0 nmol/ml) did not differ from those after normoxic conditions (26.0 ± 0.0 nmol/ml) and 60% O_2 _exposure (35.0 ± 26.0 nmol/ml), respectively.

### Activation of NF-κB and AP-1

**Figure 6:**
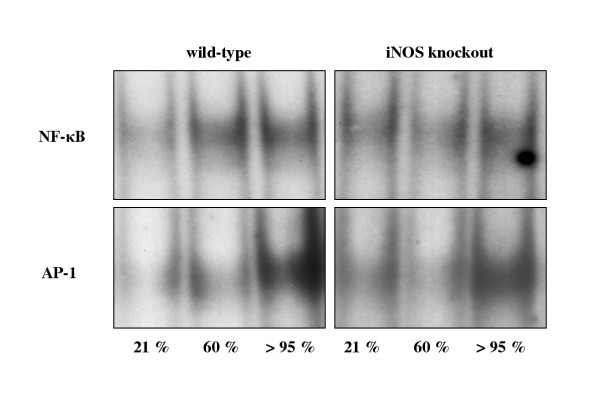
Binding activity of NF-κB and AP-1 in lung tissue from wild-type and iNOS knockout mice after 72 h normoxia or hyperoxia. Figure shown is representative for seven experiments.

In an effort to elucidate transcriptional mechanisms for increased iNOS and TNF-α expression after hyperoxic exposure, electrophoretic mobility shift assays for NF-κB and AP-1 were performed (figure 6). NF-κB and AP-1 were weakly activated under normoxic conditions. Increased activation of both NF-κB and AP-1 was observed after >95% O_2 _compared to normoxia and 60% O_2_. This enhancement of binding activity under hyperoxia appeared to be more prominent in the group of wild-type mice in comparison to iNOS knockout animals.

### iNOS mRNA expression

**Figure 7:**
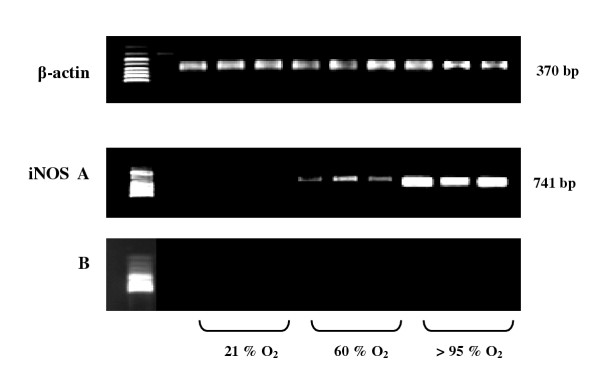
Ethidium bromide stained gels of β-actin and iNOS RT-PCR products in lung tissue from wild-type (**A**) and iNOS knockout mice (**B**) after 72 h normoxia or hyperoxia. Data shown are representative for seven experiments.

To investigate the induction of the iNOS gene in lung tissue, expression of iNOS mRNA was analyzed (figure 7). As expected, there was no expression of iNOS mRNA in lung tissues from iNOS knockout mice. In wild-type mice, hyperoxic exposure (60% and >95% O_2_) induced an increased expression of iNOS mRNA in lung samples compared to normoxic situation. Densitometric analysis was performed by determining the ratio of iNOS cDNA to β-actin cDNA. Results demonstrated a significant increase in iNOS mRNA expression upon >95% O_2 _(1.2 ± 0.1) compared to 60% O_2 _(0.8 ± 0.1).

## Discussion

Prolonged exposure to high concentrations of oxygen (>50% O_2_) during an intensive care setting to maintain arterial pO_2 _can lead to progressive lung injury. Several cellular systems including alveolar macrophages and leukocytes are involved in this process. Activation of inflammatory cells causes the release of reactive oxygen species and proinflammatory cytokines, resulting in endothelial dysfunction, tissue and alveolar edema formation, and surfactant inactivation. Furthermore, high levels of NO produced by inducible NO synthase may contribute to tissue damage. NO is directly cytotoxic or can combine with superoxide anions to form the more reactive oxidant peroxynitrite. Although a large amount of literature exists concerning the pulmonary response to oxidant exposure, some issues remain unresolved.

Our findings confirm previous results showing that hyperoxia is able to upregulate iNOS expression in lung tissue [30,34]. As expected, there was no expression of iNOS mRNA in lungs of iNOS knockout mice. In wild-type mice, exposure to 60% and >95% O_2 _induced a significant increase in iNOS mRNA expression. This enhanced iNOS mRNA expression during hyperoxic exposure seems to contradict findings reported in a study published by Arkovitz and colleagues, in which hyperoxia did not induce iNOS expression in lungs of mice [32]. This may be explained by the fact that the detection of iNOS mRNA by using northern blot technique is not as sensitive as RT-PCR. In accordance with results from others [28-30], we found little iNOS protein immunostaining under normoxic conditions and 60% oxygen exposure, while hyperoxic exposure >95% O_2 _induced a prominent expression of iNOS protein in the lungs from wild-type mice (data not shown).

The data from the present study demonstrate that *in vivo *oxygen exposure significantly elevated total BAL cell count after 72 h >95% O_2 _both in wild-type and in iNOS knockout mice. According to this, oxygen exposure resulted in a significant enhancement in the number of neutrophils and lymphocytes in BAL fluid, combined with a significant reduction in the number of alveolar macrophages both in wild-type and iNOS knockout mice. Dedhia et al. also found elevated numbers of neutrophils and lymphocytes combined with decreased numbers of alveolar macrophages in rat lungs [45]. Recent studies report that, although iNOS deficiency does not affect leukocyte rolling and adhesion following treatment with thrombin [46], iNOS-deficient mice have significantly elevated leukocyte accumulation and enhanced leukocyte-endothelium interactions in endotoxinemia [24]. These results suggest that iNOS expression plays a potent role in regulation of leukocyte recruitment depending on the way of induction. Hyperoxia-induced inflammatory cell influx, particularly of neutrophils, can contribute to oxidant stress through formation of reactive oxygen species. Auten and collaborators demonstrated that DNA damage in hyperoxia-exposed rat lungs may be reduced by blocking neutrophil influx [47]. In our model of oxidant injury, no effect of iNOS deficiency on BAL cell differentials could be made out, whereas total BAL cell counts were significantly elevated in wild-type mice compared to iNOS knockout mice. The increase in the number of neutrophils and lymphocytes in BAL fluid may partially reflect the loss of integrity of the endothelium barrier. This damage is indicated by a significant elevation of total protein concentration and LDH activity after acute hyperoxia in wild-type mice in comparison to iNOS knockout animals. Kleeberger and colleagues previously reported that iNOS expression is involved in ozone-induced lung hyperpermeability showing reduced mean BAL fluid protein and leukocyte accumulation [48].

Recent studies indicate that iNOS also plays a proinflammatory role in the development of asbestosis-related pulmonary disorders, measured as a significantly decreased total protein count, LDH activity, and nitrotyrosine staining in iNOS-deficient mice [27]. In contrast, Kobayashi et al. reported that hyperoxia caused an increased accumulation of leukocytes, elevated LDH activity and albumin concentration, and a higher wet-dry-ratio in lungs from iNOS-deficient mice compared to wild-type animals [31]. Based on their findings, these authors suggest the presence of an iNOS-independent pathway of lung nitration and injury in hyperoxia. In our study, we found that nitrosylation of proteins in the lungs of mice exposed to >95% O_2 _was attenuated in iNOS-deficient mice (data not shown). Formation of nitrotyrosine was proposed as a relatively specific marker for detecting endogenous generation of peroxynitrite. However, recent evidence indicates that alternate reactions are capable of inducing nitration of tyrosine in proteins, for example the reaction of myeloperoxidase with hydrogen peroxide. Therefore, increased nitrotyrosine staining is considered as an indicator of "increased nitrative stress" rather than a "footprint" for the formation of peroxynitrite [49,50]. Amplified formation of reactive oxygen and nitrogen species can be proved by determination of thiobarbituric acid reactive substances, a secondary product of lipid peroxidation indicating oxidative and/or nitrative stress [9]. In our study, significantly reduced formation of thiobarbituric acid reactive substances following >95% oxygen exposure was found in iNOS knockout mice, again suggesting a beneficial effect of iNOS deficiency on oxidant lung injury.

Cytokines may also play a role in oxygen toxicity. Several studies point out that TNF-α is produced during hyperoxic exposure [51,52]. Furthermore, hyperoxia induces sequential formation of pulmonary TNF-α and IL-6, which corresponds to the severity of pathological findings [12]. In our study, iNOS deficiency resulted in a significant decrease in BAL TNF-α concentration during hyperoxic exposure. Findings of Sass et al. also demonstrate that iNOS-derived NO regulates proinflammatory genes *in vivo *resulting in inflammatory liver injury in mice by stimulation of TNF-α production [53]. To investigate whether hyperoxia-induced TNF-α expression was regulated on the level of protein or mRNA, activation of the redox-sensitive transcription factors NF-κB and AP-1 was analyzed. As recently described, NF-κB was activated following hyperoxia resulting in an increase in TNF-α and IFN-γ gene expression in murine pulmonary lymphocytes [35]. Moreover, we found that the activation of both factors seen in wild-type mice was weaker in iNOS knockout mice suggesting that induction of iNOS upon hyperoxia may in fact activate these transcription factors. These findings contrast the silencing effect of NO on NF-κB demonstrated upon stimulation with LPS or silica [54]. Data from Kupatt et al. [55] also indicate a negative feedback mechanism of eNOS-derived NO on activation of NF-κB following myocardial reoxygenation. In addition to isotype-specific differences in NO forming capacity, the synergistic NF-κB and AP-1 activation upon an reactive oxygen or nitrogen species challenge might diminish the inhibitory effect of NO. Recent studies indicate that exogenously administered NO causes increased c-fos and c-jun gene and protein expression combined with an evident AP-1 binding activity mediated by reactive oxygen and nitrogen species [56].

## Conclusions

Taken together, our data show that the absence of the iNOS gene does attenuate, but not fully abolish, oxidation, nitration, and cytotoxicity in response to acute hyperoxic exposure. The degree of transcriptional activation, inflammation, and oxidative lung injury caused by hyperoxia is significantly reduced in iNOS knockout mice compared to wild-type animals. In conclusion, these findings provide evidence to suggest that, upon hyperoxic exposure to >95% O_2_, proinflammatory effects of iNOS may be predominant, thereby contributing to the extent of acute hyperoxic lung injury.

## Authors' contributions

AKH carried out the hyperoxic model, subsequent cytological and biochemical analyses, and writing and preparation of the manuscript. MD and FK participated in the direction of the study as well as in writing and preparation of the manuscript. CK carried out the electrophoretic mobility shift assays. The data presented in this paper are part of the doctoral thesis of AKH. All authors read and approved the final manuscript.

## List of abbreviations

AP-1 activator protein-1

BAL bronchoalveolar lavage

iNOS inducible nitric oxide synthase

LDH lactate dehydrogenase

LPS lipopolysaccharide

NF-κB nuclear factor-kappa B

NO nitric oxide

TBARS thiobarbituric acid reactive substances

TNF-α tumor necrosis factor-alpha

**Figure 2:**
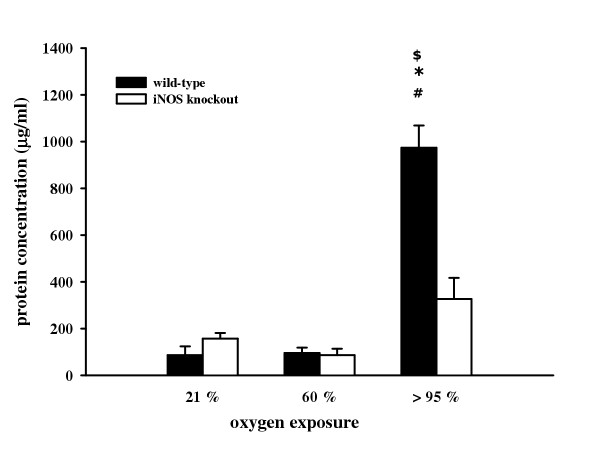
Protein concentration in BAL from wild-type and iNOS knockout mice after 72 h exposure to 21%, 60%, and >95% O_2_. Data are mean ± SEM of seven mice for each group. ^#^p < 0.05 vs. normoxia; *p < 0.05 vs. 60% O_2_; ^$^p < 0.05 vs. iNOS knockout mice.
